# A Very Unusual Presentation of Miliary Tuberculosis and Osteomyelitis as an Incidental Finding of Musculoskeletal Pain

**DOI:** 10.7759/cureus.44207

**Published:** 2023-08-27

**Authors:** Prachi B Patel, Bhavi Purohit

**Affiliations:** 1 Internal Medicine, Philadelphia College of Osteopathic Medicine, Atlanta, USA; 2 Internal Medicine, Wellstar North Fulton Hospital, Roswell, USA

**Keywords:** primary tb, spondylitis, disseminated tuberculosis, osteomyelitis, miliary tuberculosis

## Abstract

Tuberculosis (TB) is a highly infectious disease that takes the primary or latent route. In primary TB, the patient often presents with constitutional symptoms such as cough, fever, weight loss, and hemoptysis. This 83-year-old patient was sent to the emergency department (ED) by her primary care physician after abnormal imaging for acute back and neck pain. Imaging revealed pulmonary TB with possible tuberculosis osteomyelitis versus metastatic carcinoma to the cervical vertebrae. The case’s unique presentation gives light to the need for more research on miliary TB and tuberculosis spondylitis/osteomyelitis in various populations and circumstances to ensure prompt and adequate patient care.

## Introduction

To this day, tuberculosis (TB) is the leading infectious cause of death in the world, surpassing HIV/AIDS. There are over 10 million cases of TB each year and over two million corresponding deaths. This disease predominates mainly in developing countries such as India, China, Nigeria, etc., and approximately 60% of the associated deaths also come from these regions [1].

Tuberculosis is caused by Mycobacterium tuberculosis bacteria, and it is transmitted via respiratory droplets [2]. When a person first comes in contact with TB, the progression of the disease can lead to primary TB or latent TB, which can be reactivated later based on the immunity of the individual. The classic findings associated with TB include a chronic productive cough, night sweats, fever, weight loss, and hemoptysis [1].

Though pulmonary symptoms are the most common complications, TB complications arise when the infection is disseminated, meaning the disease has progressed to at least two organs in the body such as the liver, brain and spine, adrenal glands, bones, and lymphatics. Aggressive dissemination can cause miliary TB where there are both pulmonary and extrapulmonary symptoms. Of the extrapulmonary symptoms, the musculoskeletal region represents approximately 10% of the extrapulmonary symptoms and 1-5% of TB cases, and the most common area is the vertebral segments causing tubercular spondylitis/osteomyelitis [3,4]. Miliary TB is severe enough to have a mortality of about 25-30% percent of those people diagnosed with it and can lead to complications such as meningitis, encephalopathy, etc. [3].

## Case presentation

In January 2023, an 83-year-old female emigrant from China was sent to the emergency department (ED) by her primary care physician with acute back and neck pain since December 2020. Her diffuse bilateral back pain was a 7/10 severity causing difficulty with movement and walking, and her neck pain caused radicular pain in both upper extremities. Through further imaging for her acute pain, her pulmonary status came to light. Her primary care physician had ordered a CT of the chest with IV contrast and a cervical spine MRI.

**Figure 1:**
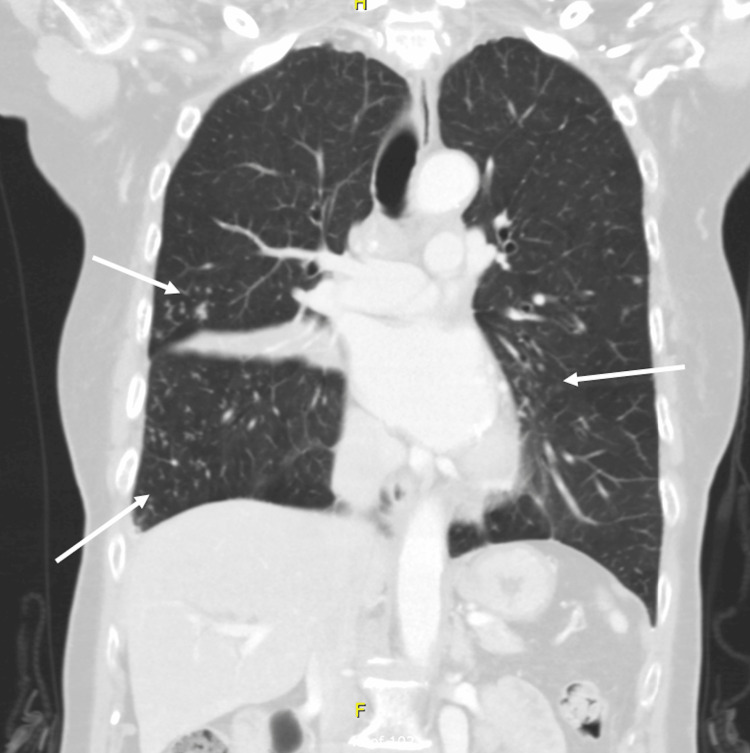
Coronal CT chest showing bilateral, multilobar, tree-in-bud opacities

**Figure 2:**
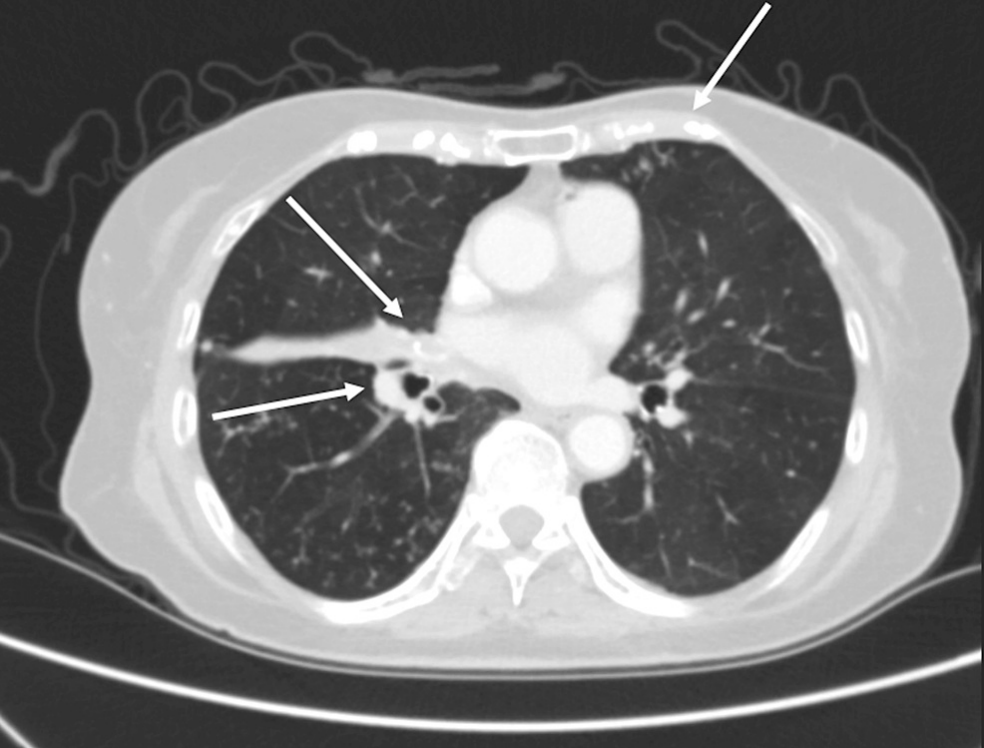
CT chest showing calcified mediastinal adenopathy, nodular pleural thickening, and bronchus obstruction in the right lung lobe

**Figure 3:**
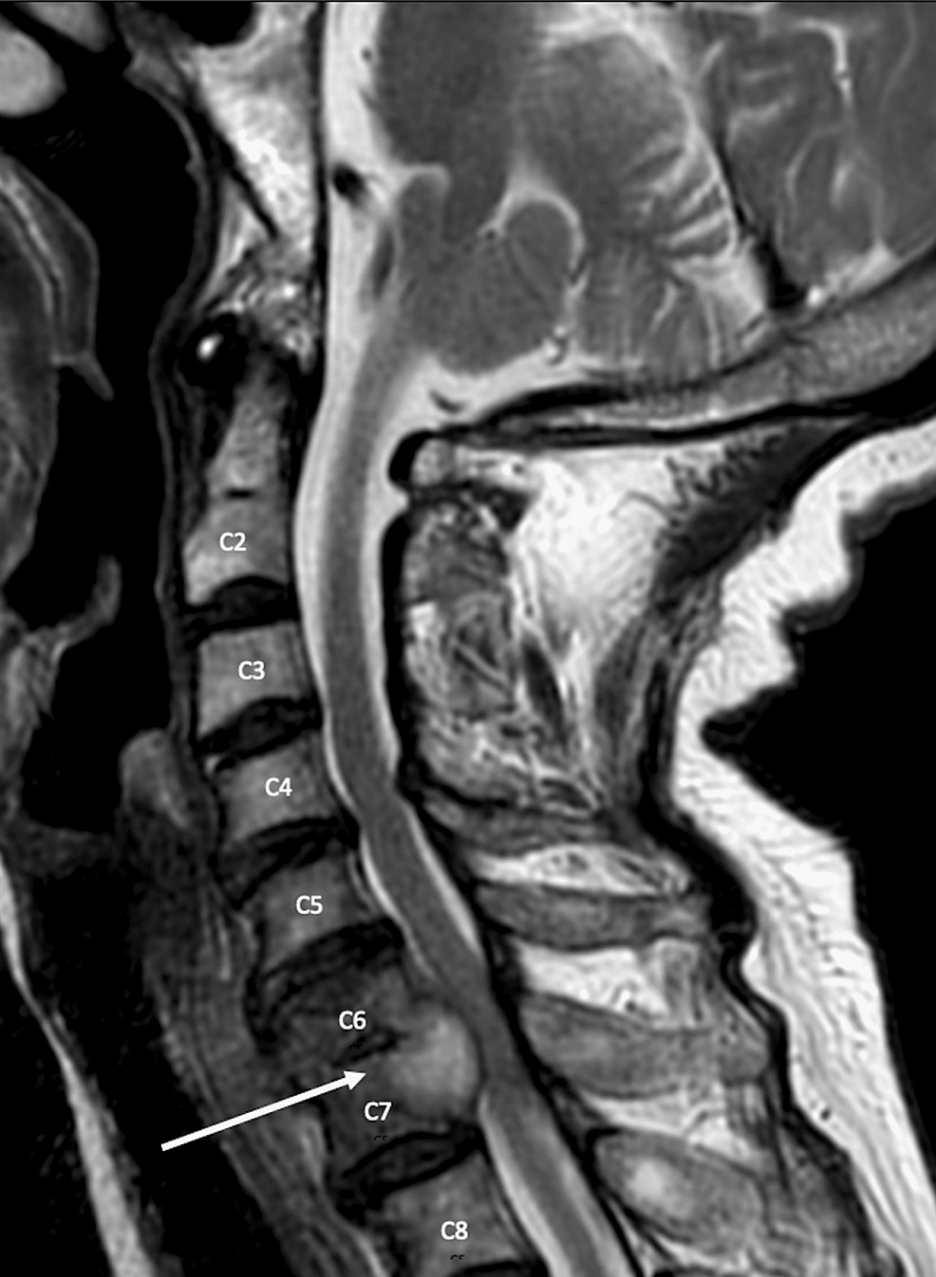
Sagittal cervical spine MRI portraying compressive collapse of the C6/C7 vertebrae, stenosis, and cord compression

**Figure 4:**
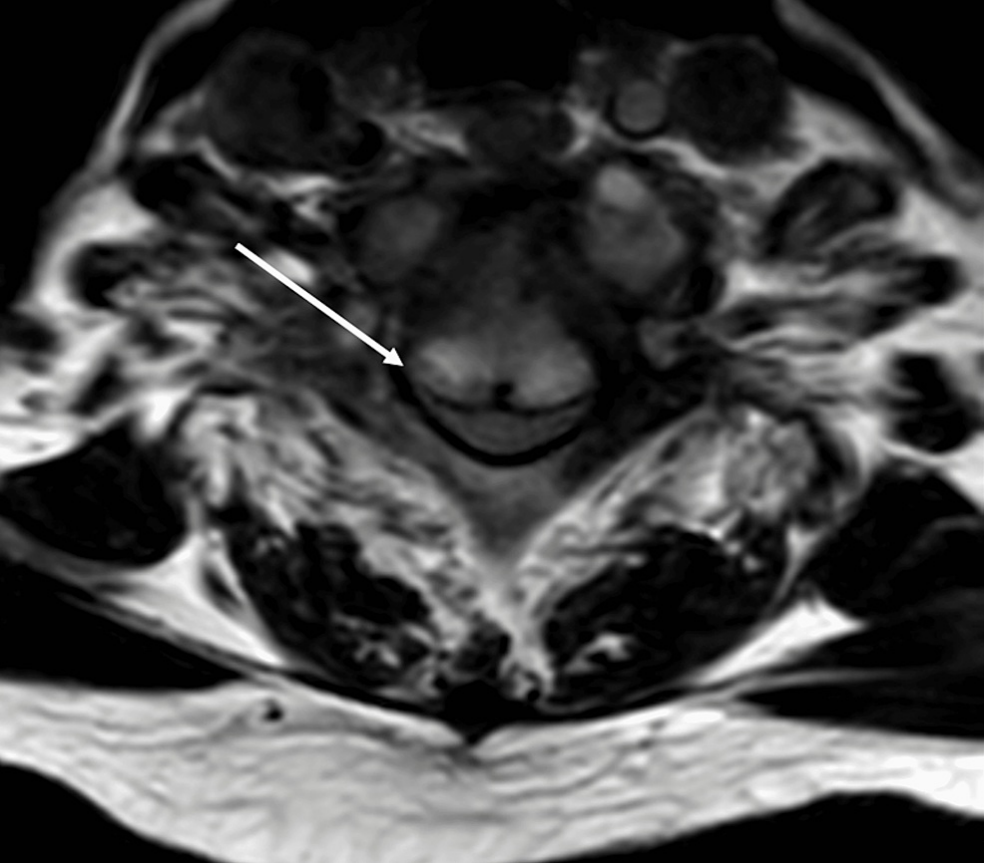
Axial cervical spine MRI portraying posterior repulsion of the C6/C7 vertebrae due to neoplasm versus infection

The CT of the chest showed the development of diffuse, multilobar, tree-in-bud opacities throughout both lungs with a few scattered superimposed focal patchy airspace opacities (Figure 1). There was partially calcified mediastinal adenopathy and nodular pleural thickening along the right lower lobe. There was also progression of the right middle lobe bronchus obstruction with a partial collapse of the right middle lobe (Figure 2). The MRI of the cervical spine portrayed abnormal compressive collapse and narrow signals involving the C6/C7 vertebrae. There was posterior retropulsion of the bone due to epidural extension of neoplasm versus infection causing severe stenosis and moderate cord compression (Figure 3, Figure 4). There was also secondary paraspinal edema with two cystic collections anterior to the C7 body. Putting both imaging studies together, the differential diagnosis for the acute back pain suggested metastatic disease versus osteomyelitis/discitis highly suspicious for infectious etiology such as pulmonary TB.

Imaging pushed the patient to be sent to the hospital, but her symptoms were unconventional for probable TB. While she did have diaphoresis during the night and chills throughout the day, she had no symptoms of hemoptysis, cough, fevers, shortness of breath, wheezing, stridor, nausea, vomiting, or headaches. Her vital signs upon admission were baseline hypertension (147/77), a normal resting heart rate of 88 beats per minute, temperature of 98.8 degrees F, a respiratory rate of 20 breaths per minute, oxygen saturation (SpO_2_) of 96%, and a body mass index of 17.58 kg/m^2^. She was clear to auscultation bilaterally, with no respiratory distress, normal pulmonary effort, and no wheezing, rales, or rhonchi. While she was a never-smoker and has not had any personal history with TB, her parents both died from TB when young.

**Figure 5:**
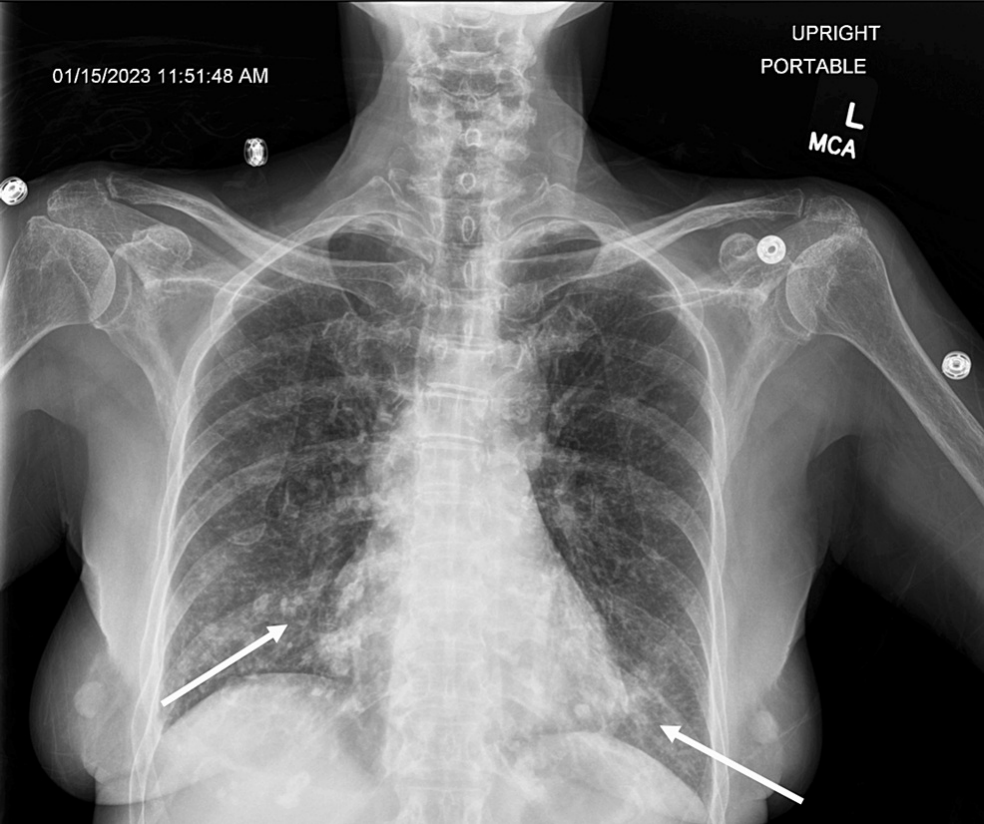
Chest X-ray revealing millet seed-sized tuberculosis foci in bilateral lungs

This patient’s ED chest X-ray provided further evidence that the several small nodular opacities within both lung fields could be compatible with miliary TB as millet seed-sized TB foci were seen in the lungs due to hematogenous spread of tubercle bacilli (Figure 5). While the Quantiferon 4T incubated was positive, the blood cultures and stains were all negative for mycobacterial and fungal organisms. Bronchoscopy then took bilateral washings and right bronchial biopsy, and both were positive for Mycobacterium tuberculosis (MTB) polymerase chain reaction (PCR) and acid-fast bacillus (AFB) stain acid-fast bacilli for Mycobacterium tuberculosis complex. The CT of the cervical spine without IV contrast depicted extraosseous extension of the disease at C6/C7, including the involvement of the ventral canal thus favoring the sequelae of infectious disease like osteomyelitis rather than neoplasm. With her osteomyelitis along with the acute TB infection, her laboratory work came back with an elevated WBC, C-reactive protein, and erythrocyte sedimentation rate (Table 1).

**Table 1:** Laboratory values

Laboratory test	Value	Reference range
White blood cell (WBC)	21.97	4.5-11.0 x 10^9^
C-reactive protein	0.6 mg/dL	<0.5 mg/dL
Erythrocyte sedimentation rate	55 mm/hr	0-29 mm/hr

This patient was diagnosed with miliary TB and started on aggressive multidrug therapy, including rifampin, isoniazid, pyrazinamide, and ethambutol for nine months. Her aggressive TB treatment began in the hospital, but she was then discharged to continue the treatment outpatient. She was followed up by Infectious Disease, her primary care physician, and the local health department to provide the appropriate medications and to monitor her symptoms.

## Discussion

Miliary TB only infects about 1-2% of all TB cases, thus showing how rare this patient’s presentation was. Furthermore, it is usually seen in a population under the age of 40 and with symptoms related to only one organ system, including the lungs, rather than this 83-year-old patient having mainly upfront extrapulmonary symptoms in the musculoskeletal system [3]. Tuberculous spondylitis classically presents with back pain in 83-100% of patients, as did this patient along with paraplegia, paraparesis, and scoliotic deformities, but it commonly infects the thoracic segments as opposed to the cervical vertebral bodies in this patient [3,4]. Research shows that the TB infection begins at the anterior vertebral body and once two vertebrae are involved, there can ultimately be vertebral narrowing and spinal cord compression along with paravertebral abscesses all seen in this patient [5].

Similar to the predicted findings of primary TB, this patient also had localization to the middle portion of the lungs known as Ghon focus, but she did not have other constitutional symptoms like chronic cough, weight loss, fever, and hemoptysis [1]. Her initial and only complaint was regarding her back and neck pain, which is very uncommon when it comes to the intensity of untreated TB. Her TB was only diagnosed secondary to further skeletal imaging. This patient had an atypical TB presentation, as she had late pulmonary symptoms and initially presented with what seemed like musculoskeletal pain. Common presentations of TB include respiratory distress in the form of shortness of breath and cough, and in approximately 30-60% of TB cases, pleural effusion is the most common extrapulmonary complication. For miliary TB specifically, it has been shown that bilateral pleural effusions should be more apparent, yet absent in this patient [6].

Neurosurgery also completed anterior C5-C7 spine cervical fusion, discectomy, and corpectomy as well as posterior C5-T2 fusion, laminectomy, and corpectomy. Surgical pathology histologically showed osteomyelitis with necrotizing granulomatous inflammation rather than metastatic carcinoma, as classic TB histological features usually present with necrotizing granuloma or central caseation [5]. What differentiates this patient from the official diagnosis of tuberculous spondylitis is that the surgical pathology was negative for mycobacterial and fungal organisms though histology supports the diagnosis of cervical infection. However, a definitive diagnosis of TB depends on the isolation of Mycobacterium; therefore, this patient with pulmonary TB would have osteomyelitis not from TB due to the negative C-arm fluoroscopy-guided percutaneous needle biopsy (PNB) with 94.1% specificity [3,4].

## Conclusions

This case is unique in its presentation along with the resulting differential diagnoses. It shows how crucial it is to not only focus on the pulmonary system and its repercussions from TB but also how other systems can be affected as TB disseminates and progresses to other parts of the body. For this patient, the bacteria manifestation was rare in that it infiltrated the nervous system and presented with a variety of uncommon nervous system symptoms before the typically known symptoms arose. It is also important to understand the various stages and types of TB in local and foreign populations of all ages to ensure there is comprehensive knowledge about this disease. With accurate, thorough, and vast research, we can create adequate management plans to allow proper patient care and decrease morbidity and mortality.
